# A phase 2 trial of trifluridine/tipiracil plus nivolumab in patients with heavily pretreated microsatellite‐stable metastatic colorectal cancer

**DOI:** 10.1002/cam4.3630

**Published:** 2021-02-05

**Authors:** Manish R. Patel, Gerald S. Falchook, Kensuke Hamada, Lukas Makris, Johanna C. Bendell

**Affiliations:** ^1^ Florida Cancer Specialists and Sarah Cannon Research Institute Sarasota Florida USA; ^2^ Sarah Cannon Research Institute at HealthONE Denver Colorado USA; ^3^ Taiho Oncology, Inc Princeton New Jersey USA; ^4^ Stathmi, Inc New Hope Pennsylvania USA; ^5^ Sarah Cannon Research Institute and Tennessee Oncology Nashville Tennessee USA

**Keywords:** chemotherapy, clinical trials, colorectal cancer, immunotherapy

## Abstract

**Background:**

Microsatellite‐stable (MSS) colorectal cancer (CRC) tends to be poorly immunogenic, with limited treatment options. In MSS CRC xenograft models, trifluridine/tipiracil (FTD/TPI) plus programed death 1 inhibitors resulted in synergistic antitumor activity and increased tumor immunogenicity. This phase 2 study evaluated FTD/TPI plus nivolumab in patients with MSS metastatic CRC.

**Methods:**

This single‐arm, safety lead‐in study used a Simon's two‐stage design (enrolling 6 patients in the safety lead‐in, proceeding to stage 2 if ≥2 of the first 15 patients achieved a partial or complete response per immune‐related response criteria [irRC] within 6 months). Patients with histologically proven MSS mCRC, and disease progression after ≥2 prior chemotherapy regimens received FTD/TPI (35 mg/m^2^ twice daily; days 1–5 and 8–12 every 28 days) plus nivolumab (3 mg/kg every 2 weeks).

**Results:**

Between August 2016 and January 2017, 18 patients (50% men; median age 56.5 years) were enrolled; 72% had colon cancer and 56% had *KRAS* mutations. All patients received treatment (median, 2.5 cycles [range, 1–8]). No dose‐limiting toxicities were observed in the study. The most frequent adverse events (AEs) of any cause and grade were nausea (67%), diarrhea (61%), and neutropenia (50%); 13 patients (72%) experienced grade ≥3 AEs. No patients discontinued treatment because of AEs. No patient achieved a tumor response (either per Response Evaluation Criteria in Solid Tumors [RECIST] or irRC), and the study did not progress to the second stage. Stable disease was achieved in 8 patients per irRC and in 10 patients per RECIST. Median progression‐free survival was 2.2 months (95% CI, 1.8–6.0 months) per irRC and 2.8 months (95% CI, 1.8–5.1 months) per RECIST.

**Conclusion:**

Patients with refractory MSS metastatic CRC failed to experience clinical benefit with FTD/TPI plus nivolumab, although safety data in this population indicated tolerability and feasibility of this combination.

**Trial registration number:**

NCT02860546.

## Graphical abstract

This phase 2 study utilized a Simon’s 2‐stage design to evaluate the combination of trifluridine/tipiracil (FTD/TPI) and nivolumab in patients with microsatellite‐stable (MSS) metastatic colorectal cancer (mCRC). Among 18 patients enrolled in the first stage, no clinical responses were observed with FTD/TPI plus nivolumab, and therefore, the study did not progress to the second stage. Patients with refractory MSS mCRC failed to experience clinical benefit with the FTD/TPI plus nivolumab combination, although safety data indicated that this combination was tolerable in these patients.

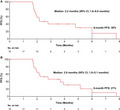

## 1 INTRODUCTION

Approximately 85% of all colorectal cancers (CRCs) are microsatellite‐stable (MSS) or mismatch‐repair proficient. Among patients with metastatic CRC (mCRC), this percentage is even higher, with only 4% to 7% of patients reported to have microsatellite‐instable (MSI) tumors.^1^, ^2^, ^3^ Patients with MSS CRC have a poorer stage‐adjusted survival and are more prone to lymph node and distant metastatic spread than those with MSI CRC.^1^, ^4^, ^5^ Treatment options are limited for patients with MSS mCRC whose disease has progressed following first‐line chemotherapy,^6^, ^7^ and benefit from later‐line therapy is short‐lived in these patients.

Immune checkpoint inhibitors, particularly those targeting the programed cell death 1 (PD‐1) pathway such as nivolumab and pembrolizumab, have demonstrated durable clinical benefit in patients with MSI CRC and are now the standard of care in these patients.^8^, ^9^, ^10^ However, patients with MSS tumors have responded poorly to anti‐PD‐1 monotherapy.^8^, ^10^ Unlike MSI tumors, MSS tumors tend to be poorly immunogenic, with high immunosuppressive activity and poor tumoral T‐cell infiltration.^11^, ^12^, ^13^ Chemotherapy has been shown to potentiate the activity of immune checkpoint inhibitors by causing immunogenic cell death, which in turn increases effector T‐cell activity and decreases immunosuppressive activity in the tumor microenvironment.^13^, ^14^ Preclinical and preliminary clinical data from ongoing trials indicate that combining immunotherapy with chemotherapy may be a feasible approach in treating patients with MSS CRC.^12^, ^13^, ^15^

Trifluridine/tipiracil (FTD/TPI) is an oral cytotoxic chemotherapeutic agent comprising trifluridine, an antineoplastic thymidine analog, and tipiracil, which prevents trifluridine degradation. FTD/TPI was shown to improve overall survival (OS) versus placebo in patients with mCRC refractory to standard therapy^7^ and demonstrated preliminary efficacy in patients with mCRC when combined with another agent such as bevacizumab.^16^ In preclinical studies with MSS CRC xenograft models, FTD/TPI combined with an anti‐PD‐1 antibody resulted in significantly greater tumor regression than either agent administered alone, which indicated synergistic activity of the combination in patients with MSS CRC.^17^ In addition, increased tumor immunogenicity (a higher cluster of differentiation [CD]8+ T‐cell to lymphocyte ratio and a lower immunosuppressive regulatory T‐cell to CD4+ T‐cell ratio) was observed with the FTD/TPI–anti‐PD‐1 combination than with either agent alone.

These data formed the basis for this single‐arm, safety lead‐in, phase 2 study, which evaluated the safety and antitumor activity of FTD/TPI in combination with nivolumab in patients with MSS mCRC refractory to standard regimens.

## 2 METHODS

### 2.1 Patients

Eligible patients were aged ≥18 years with histologically confirmed MSS metastatic or locally advanced colorectal adenocarcinoma (assessed by a local laboratory with either a prior or a fresh biopsy sample) and had ≥1 measurable lesion for Response Evaluation Criteria in Solid Tumors (RECIST) or immune response‐related criteria (irRC) assessments. In addition, eligible patients were refractory to ≥2 prior lines of standard chemotherapy (fluoropyrimidines, irinotecan, oxaliplatin, antivascular endothelial growth factor, or antiepidermal growth factor receptor therapy) and had an Eastern Cooperative Oncology Group performance status of 0 or 1. Key exclusion criteria were prior treatment with FTD/TPI or immune checkpoint inhibitors; major surgery, extended field radiation, or anticancer therapy within 2 to 4 weeks of initiating therapy; and any history of immune‐mediated reactions.

### 2.2 Study design and treatment

This was a multicenter, single‐arm, safety lead‐in, phase 2 study that used a Simon's two‐stage design.^18^ Six patients were initially enrolled in stage I and evaluated for safety and tolerability. An additional nine response‐evaluable patients were planned for enrollment in stage I if ≤1 dose‐limiting toxicity (DLT) was observed among the first six patients. An interim analysis of safety and efficacy was performed after a 6‐month follow‐up. Initiation of stage II (with enrollment of another 10 patients) was contingent on two or more of the 15 patients enrolled in stage I achieving a best overall response of partial or complete response.

Patients received FTD/TPI 35 mg/m^2^ twice daily (BID) orally on days 1–5 and 8–12 of a 28‐day cycle and intravenous nivolumab (3 mg/kg/dose) on days 1 and 15 of the 28‐day cycle. In the event of toxicity, the FTD/TPI dose could be reduced in 5‐mg/m^2^ increments to a minimum dose of 20 mg/m^2^ BID, but nivolumab dose reductions were not permitted. Treatment was administered until disease progression by irRC, unacceptable toxicity, patient request, or a physician's decision to withdraw treatment.

### 2.3 Study objectives and statistical considerations

The primary objective of the study was to estimate the immune‐related objective response rate (irORR) in patients treated with FTD/TPI plus nivolumab. Secondary objectives included determination of the phase 2 dose of the combination regimen, safety, ORR per RECIST v1.1, progression‐free survival (PFS; per irRC or RECIST), disease control rate, and OS. Exploratory objectives included examining association of microsatellite status, programed death ligand 1 (PD‐L1) positivity, and tumor‐infiltrating lymphocytes with response and toxicity.

Sample‐size considerations were based on the Simon's two‐stage design (described above), with an irORR of ≤10% considered unacceptable at an approximate 5% one‐sided significance level and 80% power. Assuming 10% to 15% nonevaluability for DLT and/or irRC assessments, a total of 30 to 35 patients were targeted for enrollment into the study.

### 2.4 Efficacy assessments

All patients who received ≥1 dose of study drug and completed ≥6 months of tumor follow‐up (barring death or disease progression) were evaluable for efficacy. Tumor assessments were performed at baseline and after every two cycles of treatment with contrast‐enhanced computed tomography of the chest and abdomen. Response was assessed with RECIST v1.1 and irRC.

### 2.5 Safety assessments

All patients who received ≥1 dose of study drug were evaluable for safety. Patients were monitored for safety from the first dose until 30 days after the last dose or until initiation of a new anticancer treatment. Adverse events (AEs) were graded according to the National Cancer Institute Common Terminology Criteria for Adverse Events version 4.03.

A DLT was defined as ≥1 of the following FTD/TPI‐related AEs occurring during the first treatment cycle: grade 4 neutropenia lasting >7 days or febrile neutropenia, grade 4 thrombocytopenia, grade 3/4 nonhematologic toxicity, and grade 3/4 nausea/vomiting/diarrhea lasting >48 hours. In addition, any drug‐related toxicity resulting in >2 weeks’ delay in initiating cycle 2 or preventing completion of ≥80% of the planned dose administration of either drug in cycle 1 was considered a DLT.

### 2.6 Patient and public involvement statement

This research was done without patient involvement. Patients were not invited to comment on the study design and were not consulted to develop patient‐relevant outcomes or interpret the results. Patients were not invited to contribute to the writing or editing of this document for readability or accuracy.

## 3 RESULTS

### 3.1 Patient population

A total of 18 patients were enrolled in stage I of the study (from August 29, 2016, to January 26, 2017) across three sites in the United States and received ≥1 dose of study drug. Following an interim analysis, the study was halted and no patient was enrolled into stage II. At the time of data cutoff (November 3, 2017), all patients had discontinued treatment because of disease progression.

Nine of 18 patients (50%) were male, and median age was 56.5 years; 13 patients (72%) had colon cancer, and 10 patients (56%) had tumors with *KRAS* mutations. All patients had received prior systemic therapy, and most (83%) had undergone prior surgery (Table 1).

**TABLE 1:** Patient baseline characteristics and prior therapy

Characteristic	FTD/TPI plus nivolumab (N = 18)
Median age (range), y	56.5 (40–70)
Men, n (%)	9 (50)
Race, n (%)	
White	12 (67)
Black	4 (22)
Not collected	2 (11)
ECOG PS, n (%)	
0	4 (22)
1	14 (78)
Primary disease site, n (%)	
Colon	13 (72)
Rectum	3 (17)
Colorectal	2 (11)
Number of metastatic sites, n (%)	
1–2	9 (50)
≥3	9 (50)
Mutational status at baseline, n (%)	
*RAS* mutant	10 (56)
*KRAS* mutant	10 (56)
*NRAS* mutant	2 (11)
*BRAF* mutant^a^	1 (6)
Prior systemic treatment for metastatic disease, n (%)	18 (100)
Fluoropyrimidine	17 (94)
Platinum	14 (78)
Irinotecan	16 (89)
Leucovorin	16 (89)
Anti‐VEGF	16 (89)
Anti‐EGFR	7 (39)
Aflibercept	3 (17)
Other	2 (11)
Prior surgery,^b^ n (%)	15 (83)

Abbreviations: ECOG PS, Eastern Cooperative Oncology Group performance status; EGFR, epidermal growth factor receptor; FTD/TPI, trifluridine/tipiracil; VEGF, vascular endothelial growth factor.

^a^ *BRAF* status was unknown or missing for nine patients.

^b^ Related to colorectal cancer.

### 3.2 Safety

Patients received a median of 2.5 cycles (range, 1–8) of FTD/TPI plus nivolumab, and the median duration of FTD/TPI treatment was 13.4 weeks. The median relative dose intensity (ratio of the dose administered to the planned dose) was 0.84 for FTD/TPI and 1.00 for nivolumab.

No DLTs were reported in the study. In the overall population (N = 18), the most common AEs of any cause were nausea (67%); diarrhea (61%); neutropenia (50%); abdominal pain, fatigue, and vomiting (33% each); and anemia (28%; Table 2). Grade ≥3 AEs of any cause were reported in 13 patients (72%), most commonly neutropenia (28%); diarrhea (17%); and abdominal pain, anemia, fatigue, and nausea (11% each). Grade ≥3 AEs were considered related to FTD/TPI in 10 patients (56%) and to nivolumab in five patients (28%). Grade 4 AEs of any cause were reported in two patients (neutropenia [n = 1] and decreased neutrophil count [n = 1]). No grade 5 AE or treatment‐related death was reported in the study. AEs of any cause led to FTD/TPI dosing modifications (dosing delays or interruptions) in 11 patients (61%) and to nivolumab dosing modifications in three patients (17%). These nivolumab dosing modifications were all dosing delays or interruptions, and all three patients had concomitant FTD/TPI dosing modifications. One patient skipped one nivolumab dose because of grade 2 anorexia and grade 3 weakness, and two patients had nivolumab dose interruptions due to diarrhea (n = 1) and grade 2 increased bilirubin (n = 1). No patient discontinued study treatment because of toxicities.

**TABLE 2:** AEs in patients receiving FTD/TPI plus nivolumab

AE	FTD/TPI plus nivolumab (N = 18)	
	Any grade, n (%)	Grade ≥3, n (%)
Any AE of any cause	18 (100)	13 (72)
Any treatment‐related AE		
Related to FTD/TPI	18 (100)	10 (56)
Related to nivolumab	16 (89)	5 (28)
Related to both	15 (83)	4 (22)
AEs of any cause in ≥10% of patients		
Nausea	12 (67)	2 (11)
Diarrhea	11 (61)	3 (17)
Neutropenia^a^	9 (50)	5 (28)
Abdominal pain	6 (33)	2 (11)
Fatigue	6 (33)	2 (11)
Vomiting	6 (33)	1 (6)
Anemia	5 (28)	2 (11)
Constipation	4 (22)	0
Pruritus	4 (22)	0
Decreased appetite	3 (17)	0
Dyspnea	3 (17)	0
Pyrexia	3 (17)	1 (6)
Urinary‐tract infection	3 (17)	1 (6)
Asthenia	2 (11)	1 (6)
Dysuria	2 (11)	0
Follicular rash	2 (11)	0
Hypertension	2 (11)	1 (6)
Increased blood bilirubin	2 (11)	1 (6)
Maculopapular rash	2 (11)	0
Mucosal inflammation	2 (11)	1 (6)
Nasal congestion	2 (11)	0
Rhinorrhea	2 (11)	0
Stomatitis	2 (11)	0
Thrombocytopenia	2 (11)	0
Upper abdominal pain	2 (11)	0
Upper respiratory‐tract infection	2 (11)	0

Abbreviations: AE, adverse event; FTD/TPI, trifluridine/tipiracil.

^a^ Includes decreased neutrophil count.

### 3.3 Efficacy

**FIGURE 1:**
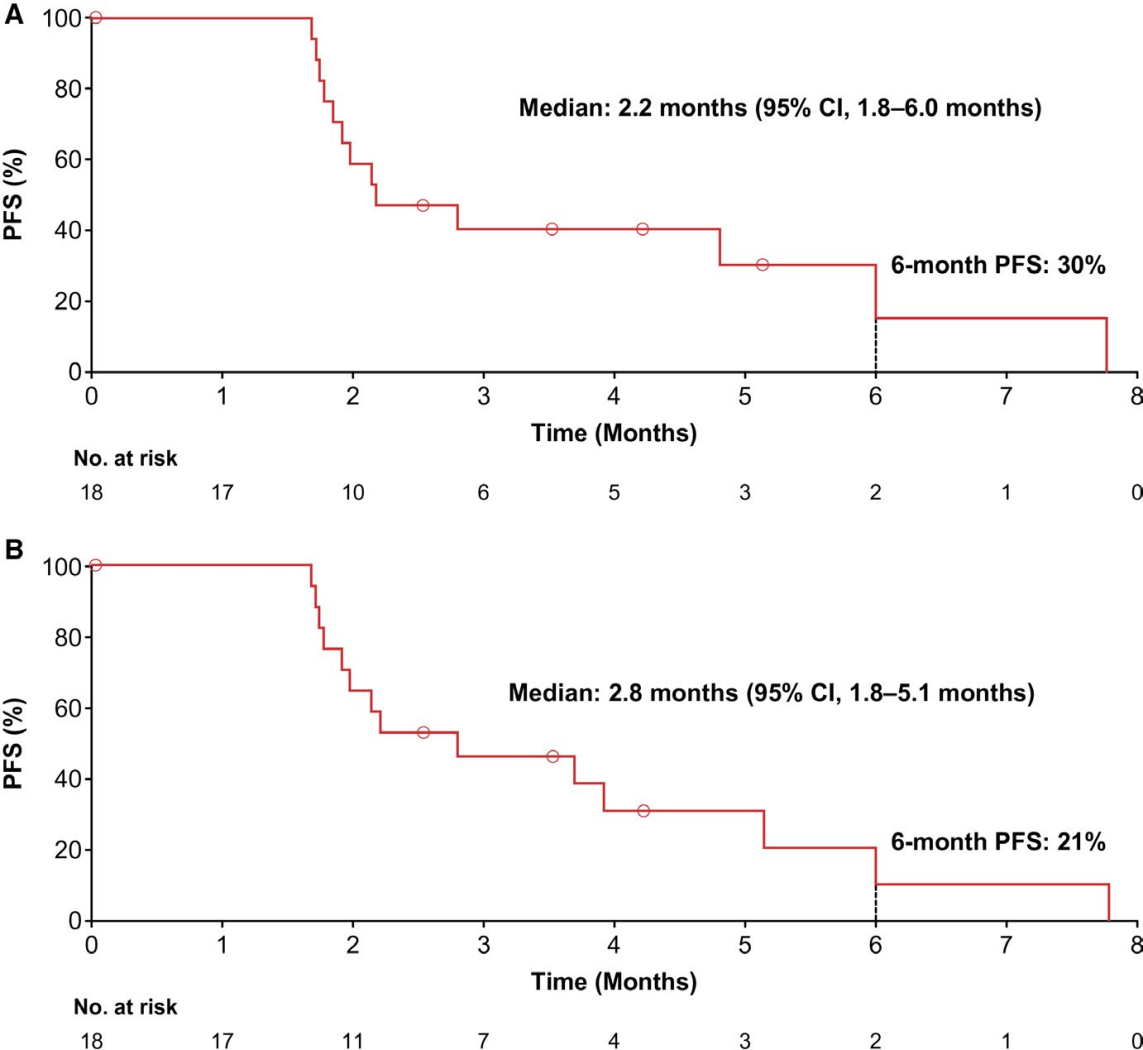
Radiologic PFS with FTD/TPI plus nivolumab. PFS was defined as the time from the first dose of study drug to disease progression as assessed per (A) irRC criteria or (B) RECIST v1.1. irRC, immune‐related response criteria; PFS, progression‐free survival; RECIST v1.1, Response Evaluation Criteria in Solid Tumors version 1.1.

Among the 18 patients enrolled in stage I, no patient experienced an objective response (partial or complete) per either irRC or RECIST (Table 3). Therefore, per the study design, the trial was stopped and no patient was enrolled into stage II. Eight patients (44%) experienced a best overall response of stable disease per irRC (10 patients [56%] per RECIST). Median radiologic PFS was 2.2 months (95% confidence interval [CI], 1.8–6.0 months) per irRC and 2.8 months (95% CI, 1.8–5.1 months) per RECIST, with respective 6‐month PFS rates of 30% and 21% (Figure 1).

**TABLE 3:** Best overall response

Response	Per irRC (N = 18)	Per RECIST (N = 18)
Best overall response, n (%)		
Complete or partial response	0	0
Stable disease	8 (44)	10 (56)
Progressive disease	7 (39)	5 (28)
Not evaluable	3 (17)	3 (17)
Disease control rate, % (95% CI)	44 (22–69)	56 (31–78)

Abbreviations: CI, confidence interval; irRC, immune response‐related criteria; RECIST, Response Evaluation Criteria in Solid Tumors.

## 4 DISCUSSION

This phase 2 study evaluating the combination of FTD/TPI plus nivolumab in patients with refractory MSS mCRC did not meet its primary endpoint. No response was observed among the 18 patients treated in stage I of the Simon's two‐stage design, and the trial was stopped for futility; no patient was enrolled into stage II of the study. The dose selection in this study was based on the recommended FTD/TPI dosing regimen (35 mg/m^2^ BID on days 1–5 and 8–12 of a 28‐day cycle) already established in the mCRC patient population^7^, ^19^ and the standard nivolumab dosing regimen (3 mg/kg every 2 weeks) used in MSI mCRC and other tumor types.^20^ This nivolumab dosing schedule also aligned well with the recommended FTD/TPI dosing schedule. The combination dosing regimen was considered optimal because no dose‐limiting toxicities or longer term toxicities were observed in this study. The combination of FTD/TPI plus nivolumab was tolerable in these patients, and AEs associated with the combination were manageable; no grade 5 event or AE‐related discontinuation was reported. The most common AEs observed (hematological and gastrointestinal in nature) were consistent with observations from prior reports of FTD/TPI treatment. The addition of nivolumab did not appear to worsen the safety profile of FTD/TPI.

The failure of the FTD/TPI plus nivolumab combination regimen to achieve clinical responses in this study further highlights the differences in biology between MSS and MSI CRC. MSI tumors are highly sensitive to immunotherapy because of their high mutational burden, which results in a high proportion of mutant neoantigens in the tumor microenvironment. As a result, these tumors are highly immunogenic, have high PD‐L1 expression, and consequently exhibit robust cytotoxic T‐cell responses.^21^ However, MSS tumors are mostly resistant to anti‐PD‐1 monotherapy because of low mutational burden, immunoexclusion, and immunosuppression.^13^ The addition of chemotherapy, antiangiogenic agents, or MEK inhibitors is thought to sensitize MSS tumors to immunotherapy, as these agents potentiate the immune response.^14^, ^22^ Despite promising preclinical evidence of synergistic activity with the FTD/TPI plus anti‐PD‐1 combination in MSS CRC,^17^ the addition of FTD/TPI was insufficient to potentiate the activity of nivolumab in our study and the response rates and median PFS observed were similar to those previously reported with anti‐PD‐1 monotherapy in this patient population.^8^, ^9^

Chemotherapy–immunotherapy combinations appear to demonstrate greater clinical activity in patients with treatment‐naïve MSS CRC: a 53% ORR was observed in a phase 2 study evaluating leucovorin/5‐fluorouracil/oxaliplatin (FOLFOX) plus pembrolizumab in 30 patients with treatment‐naïve CRC (predominantly MSS).^23^ Promising results were also obtained with other immunotherapy combination regimens in this patient population.^24^, ^25^

However, results of immunotherapy combination trials in the refractory MSS CRC population have been conflicting. A phase 1/1b study evaluating the combination of atezolizumab, an anti‐PD‐L1 antibody, and cobimetinib, an MEK inhibitor, reported objective responses in seven of 84 patients with chemorefractory mCRC; six of the responders had MSS mCRC.^26^ However, the randomized phase 3 IMblaze370 trial that evaluated atezolizumab with or without cobimetinib versus the multikinase inhibitor regorafenib in 363 patients with chemorefractory mCRC (more than 90% of patients had MSS tumors) failed to meet its primary endpoint.^27^ In this study, no improvement in OS or PFS was observed in the atezolizumab or atezolizumab plus cobimetinib arms compared with regorafenib, and ORRs ranged from 2% to 3% in the three arms. In the phase 2 CheckMate 142 study, only one response was seen among 20 patients with chemorefractory MSS mCRC treated with nivolumab plus ipilimumab (an anti‐cytotoxic T‐lymphocyte antigen‐4 antibody); in addition, severe toxicities were observed.^9^ It should be noted, however, that these data, for the most part, are preliminary and derived from small numbers of patients. The direction for combination approaches in this patient population is likely to be clarified once the results of ongoing trials evaluating other immunotherapy combination regimens in refractory MSS mCRC^12^, ^13^, ^15^ become available.

In summary, patients with refractory MSS mCRC failed to experience clinical benefit with the FTD/TPI plus nivolumab combination, although safety data indicated that this combination was tolerable in these patients. These results highlight the need for novel treatment approaches in MSS CRC, as the unmet medical need remains high for patients with refractory MSS mCRC. Preliminary results of ongoing trials indicate that other combination therapy regimens may be worth exploring in this patient population.

## 5 ETHICS APPROVAL

The study protocol and informed consent forms at the Sarah Cannon Research Institute sites were approved by the IntegReview Institutional review board (Approval ID: IORG0000689). The IRB was organized and functioned in accordance with the United States Code of Federal Regulations (CFR) (Title 21 CFR, Part 56.107 through 56.115) and/or Chapter 3 of the International Council for Harmonisation (ICH) E6 Guidelines.

## CONFLICT OF INTERESTS

MRP reports speakers bureau honoraria from Taiho Oncology and reports institutional funding from Acerta Pharma, ADC Therapeutics, Agenus, Aileron Therapeutics, AstraZeneca, Bicycle Therapeutics, BioNTech, Boehringer Ingelheim, Calithera, Celgene, Checkpoint Therapeutics, Ciclomed, Clovis, Curis, Cyteir Therapeutics, Daiichi Sankyo, Effector Therapeutics, Eli Lilly, EMD Serono, Evelo Biosciences, Forma Therapeutics, Genentech/Roche, Gilead, GlaxoSmithKline, H3 Biomedicine, Hengrui, Hutchinson MediPharma, Ignyta, Incyte, Jacobio, Janssen, Jounce Therapeutics, Klus Pharma, Kymab, Loxo Oncology, LSK Biopartners, Lycera, Macrogenics, Merck, Millennium Pharmaceuticals, Mirati Therapeutics, ModernaTX, Pfizer, Phoenix Molecular Designs, Placon Therapeutics, Portola Pharmaceuticals, Prelude Therapeutics, Qilu Puget Sound Biotherapeutics, Revolution Medicines, Ribon Therapeutics, Seven and Eight Biopharmaceuticals, Syndax, Synthorx, Stemline Therapeutics, Taiho, Takeda, Tesaro, TopAlliance, Vedanta, Verastem, Vigeo, and Xencor. GF reports institutional research funding from 3‐V Biosciences, Abbisko, AbbVie, ADC Therapeutics, Aileron, American Society of Clinical Oncology, Amgen, ARMO, AstraZeneca, BeiGene, Bioatla, Biothera, Celldex, Celgene, Ciclomed, Curegenix, Curis, Cyteir, Daiichi, DelMar, eFFECTOR, Eli Lilly, EMD Serono, Epizyme, Exelixis, Fujifilm, Genmab, GlaxoSmithKline, Hutchison MediPharma, Ignyta, Incyte, Jacobio, Jounce, Kolltan, Loxo, MedImmune, Millennium, Merck, miRNA Therapeutics, National Institutes of Health, Novartis, OncoMed, Oncorus, Oncothyreon, Poseida, Precision Oncology, Prelude, Regeneron, Rgenix, Ribon, Strategia, Syndax, Taiho, Takeda, Tarveda, Tesaro, Tocagen, Turning Point Therapeutics, U.T. MD Anderson Cancer Center, Vegenics,and Xencor; speakers bureau honoraria from Total Health Conferencing and Rocky Mountain Oncology Society; travel fees from Bristol‐Myers Squibb (2015), EMD Serono (2011, 2012, 2013), Fujifilm (2018), Millennium (2013), Sarah Cannon Research Institute; advisory role at EMD Serono, and royalties from Wolters Kluwer. KH is a salaried employee at Taiho Oncology and reports personal fees from Taiho Oncology during the study and outside the submitted work. LH is a paid consultant to and reports personal fees from Taiho Oncology during the study and outside the submitted work. JCB reports institutional research funding from Gilead, Genentech/Roche, Bristol‐Myers Squibb, Five Prime, Lilly, Merck, MedImmune, Celgene, EMD Serono, Taiho, Macrogenics, GlaxoSmithKline, Novartis, Oncomed, LEAP, TG Therapeutics, AstraZeneca, Boehringer Ingelheim, Daiichi Sankyo, Bayer, Incyte, Apexigen, Koltan, SynDevRex, Forty Seven, AbbVie, Array, Onyx, Sanofi, Takeda, Eisai, Celldex, Agios, Cytomx, Nektar, ARMO, Boston Biomedical, Ipsen, Merrimack, Tarveda, Tyrogenex, Oncogenex, Marshall Edwards, Pieris, Mersana, Calithera, Blueprint, Evelo, FORMA, Merus, Jacobio, Effector, Novocare, Arrys, Tracon, Sierra, Innate, Arch Oncology, Prelude Therapeutics, Unum Therapeutics, Vyriad, Harpoon, ADC, Amgen, Pfizer, Millennium, Imclome, Acerta Pharma, Rgenix, Bellicum, Gossamer Bio, Arcus Bio, Seattle Genetics, Tempest Tx, Shattuck Labs, Synthorx, Revolution Medicines, Bicycle Therapeutics, Zymeworks, Relay Therapeutics; institutional payment for consulting services from Gilead, Genentech/Roche, Bristol‐Myers Squibb, Five Prime, Lilly, Merck, MedImmune, Celgene, Taiho, Macrogenics, GlaxoSmithKline, Novartis, OncoMed, LEAP, TG Therapeutics, AstraZeneca, Boehringer Ingelheim, Daiichi Sankyo, Bayer, Incyte, Apexigen, Array, Sanofi, Agios, ARMO, Ipsen, Merrimack, Oncogenex, Evelo, FORMA, Innate, Arch Oncology, Prelude Therapeutics, Amgen, Seattle Genetics, Bicycle Therapeutics, Relay Therapeutics, Phoenix Bio, Cyteir, Molecular Partners, Torque, Tizona, Janssen, Tolero, TD2 (Transitional Drug Development), Moderna Therapeutics, Tanabe Research Laboratories, Beigene, and Continuum Clinical.

## AUTHOR CONTRIBUTIONS

MRP, KH, LM, and JCB designed the trial, interpreted the data, analyzed the results, and wrote the manuscript. GF was involved in trial conception, patient care, and follow‐up, as well as manuscript review and revision. KH and LM contributed to statistical analysis, data assessment/acquisition, and manuscript review. All authors read and approved the final manuscript.

## Data Availability

Data generated or analyzed during this study are on file with Taiho Oncology, Inc., and Taiho Pharmaceuticals Co., Ltd., and are not publicly available. Inquiries about data access should be sent to th-datasharing@taiho.co.jp.
